# Supplementary material for: Nanocrystallites Modulate Intermolecular Interactions in Cryoprotected Protein Solutions
Source: J Phys Chem B. 2023 Jul 3;127(27):6197–204. doi: 10.1021/acs.jpcb.3c02413 (PMC10350957; doi:10.1021/acs.jpcb.3c02413)
Supplement: Supplementary file 1 — jp3c02413_si_001.pdf [file jp3c02413_si_001.pdf]

SUPPORTING INFORMATION: Nanocrystallites Modulate Intermolecular Interactions in Cryoprotected Protein Solutions

Mariia Filianina,[*,†] Maddalena Bin,[†] Sharon Berkowicz,[†] Mario Reiser,[†] Hailong Li,[†,‡] Sonja Timmermann,[¶] Malte Blankenburg,[§] Katrin Amann-Winkel,[†,‡,‖] Christian Gutt,[¶] and Fivos Perakis[*,†]

†*Department of Physics, AlbaNova University Center, Stockholm University, S-106 91 Stockholm, Sweden*

‡*Max Plank Institute for Polymer Research, Ackermannweg 10, 55128 Mainz, Germany*

¶*Department of Physics, Universität Siegen, Walter-Flex-Strasse 3, 57072 Siegen, Germany*

§*Deutsches Elektronen-Synchrotron (DESY), Notkestrasse 85, 22607 Hamburg, Germany*

‖*Institute of Physics, Johannes Gutenberg University, 55128 Mainz, Germany*

E-mail: mariia.filianina@fysik.su.se; f.perakis@fysik.su.se

Measurement protocol

Figure S1 illustrates an example of the measurement protocol. Panels A and B represent cooling and heating which together comprise one full temperature cycle. For cooling, the temperature was varied with the rate of 4 K/min from $T = 300$ K to the target temperature $T = 195$ K. SAXS/WAXS scattering data were acquired simultaneously with an exposure time of 1 s followed by a 9 s dead time until the next measurement, i.e. every 10 s. In total, 165 scans were taken during cooling equally spanned over the entire temperature range, hence limiting the total exposure time of the sample to 165 s. The measurements during heating were started right away and the same protocol was employed. Thus, for the whole temperature cycle the sample was exposed to X-rays for 330 s.

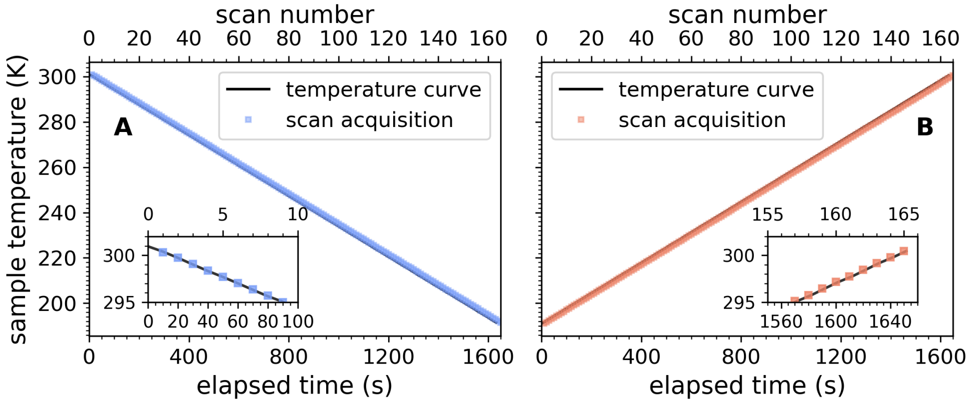

Figure S1: The measurement protocol for the entire temperature cycle including cooling (panel A) and heating (panel B). The temperature was varied with a constant rate of 4 K/min (black solid line) while the SAXS/WAXS scattering data (blue markers) were acquired simultaneously every 10 seconds, i.e. with the exposure time of 1 s and the dead time of 9 s. The insets show a zoomed-in region demonstrating the time structure of the measurements along the temperature variation line.

Radiation damage assessment

The data discussed in the main text were measured for all samples using the X-ray flux $F = 1.25 \times 10^{10}$ ph/s, where no X-ray-induced damage to the system was observed in

contrast to higher fluxes (Fig. S2).

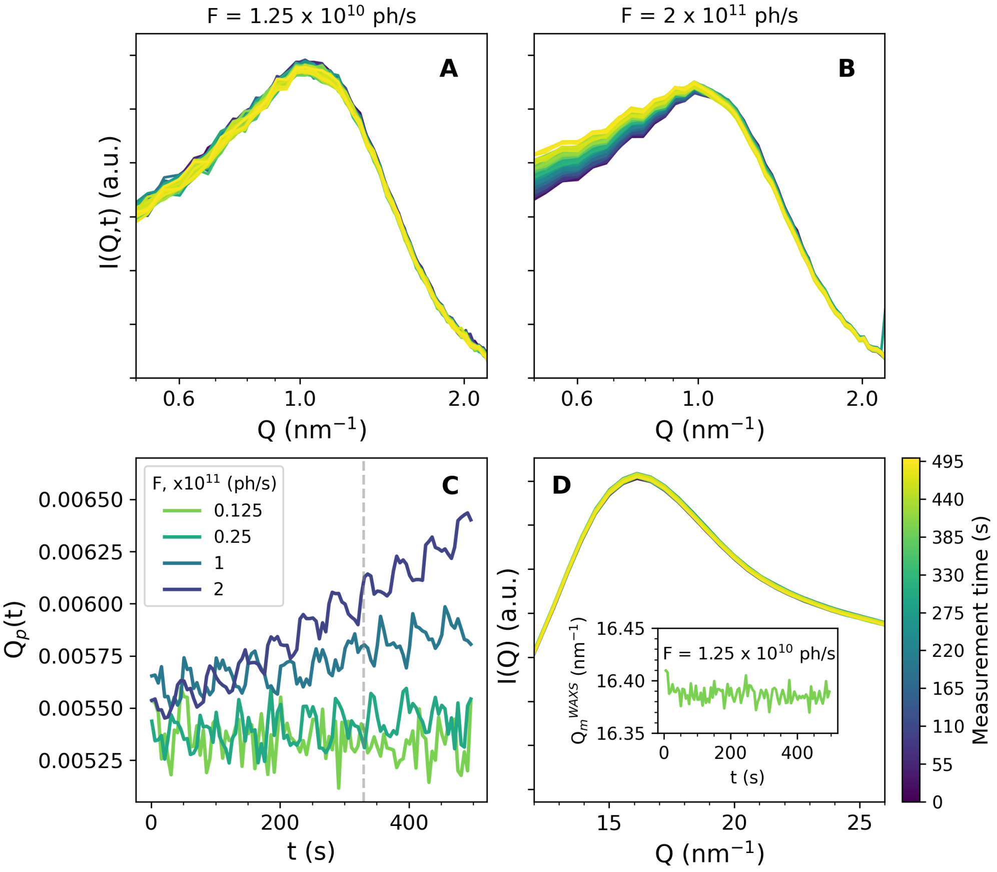

Figure S2: The SAXS intensity, $I(Q,t)$, measured using the flux $F = 1.25 \times 10^9$ ph/s (panel A) and $F = 2 \times 10^{11}$ ph/s (panel B) for 200 mg/ml lysozyme in 23 mol% glycerol-water solution at room temperature ($T = 300$ K). The color indicates the measurement time, t. (C) Porod invariant, $Q_p(t)$, calculated from the data displayed in A and B and two additional fluxes as a function of measurement time, t, i.e. the time the sample is exposed to X-rays. The vertical dashed line shows the total exposure time needed for the full temperature cycle. (D) The WAXS intensity measured using the flux $F = 1.25 \times 10^{10}$ ph/s for lysozyme in 23 mol% glycerol-water solution at room temperature ($T = 300$ K). The inset shows the absent variation of the Q-value of the first peak over the measurement time. Note that the oscillations of the integrated intensity visible in panel C for higher fluxes arise from the top-up mode of the storage ring.

Shown in Fig. S2 A evolution of the SAXS signal measured over the time span of 500 seconds using the X-ray flux $F = 1.25 \times 10^{10}$ ph/s does not exhibit any change in the Q-range of interest. On the contrary, when measured using a much higher flux ($F = 2 \times 10^{11}$ ph/s) a clear increase of $I(Q,t)$ at Q-values below 1 nm^{-1} is seen in Fig. S2 B. Here, the Porod invariant, calculated as $Q_p(t) = \int_{Q_{min}}^{Q_{max}} Q^2 I(Q,t)\,\mathrm{d}Q$, where $Q_{min} = 0.5$ nm^{-1} and

$Q_{max} = 1$ nm^{-1} is used to quantify the X-ray-induced changes in SAXS. The variation of the Porod invariant as a function of measurement time, t, is shown in Fig. S2 C for the different X-ray fluxes. It is evident that already at $F = 2.5 \times 10^{10}$ ph/s, the X-ray-induced changes in SAXS are negligible over the time span required for the full temperature cycle (330 s, dashed vertical line), discussed in the main text.

Figure S2D presents the evolution of the WAXS signal measured using $F = 1.25 \times 10^{10}$ ph/s over 500 s time span, where no indication of the X-ray-induced effect can be seen. Neither the lineshape of the scattering intensity peak, nor the position of the first maximum, Q_m^{WAXS}, plotted in the inset as a function of the measurement time, show any measurable variation.

Temperature dependent WAXS for dilute lysozyme solution

Figure S3 shows the variation of the WAXS scattering intensities measured for 10 mg/ml lysozyme in glycerol-water solution upon cooling down to 195 K (panel A) and warming back up to 300 K (panel B). The overall behavior is similar to that observed for the concentrated lysozyme solution discussed in the main text. Specifically, small ice Bragg peaks develop upon reheating the sample while they are not visible upon cooling down, as seen in Figs. S3 E,F, which focuses on the temperature evolution of the ice $I_h[101]$ diffraction peak highlighted by the white rectangle in Fig. S3 C. Based on the peak's width, the sizes of the ice nanocrystallites are in the order of 15 nm, i.e. similar to those formed in 200 mg/ml lysozyme in glycerol-water solution.

Furthermore, the temperature dependence of the first WAXS peak Q position for the dilute lysozyme solution is qualitatively similar to that observed for the concentrated one, shown for comparison in Figs. S4 A,B. A change of the linear slope at $T \approx 245$ K upon cooling as well as a thermal hysteresis upon reheating are observed for 10 mg/ml lysozyme

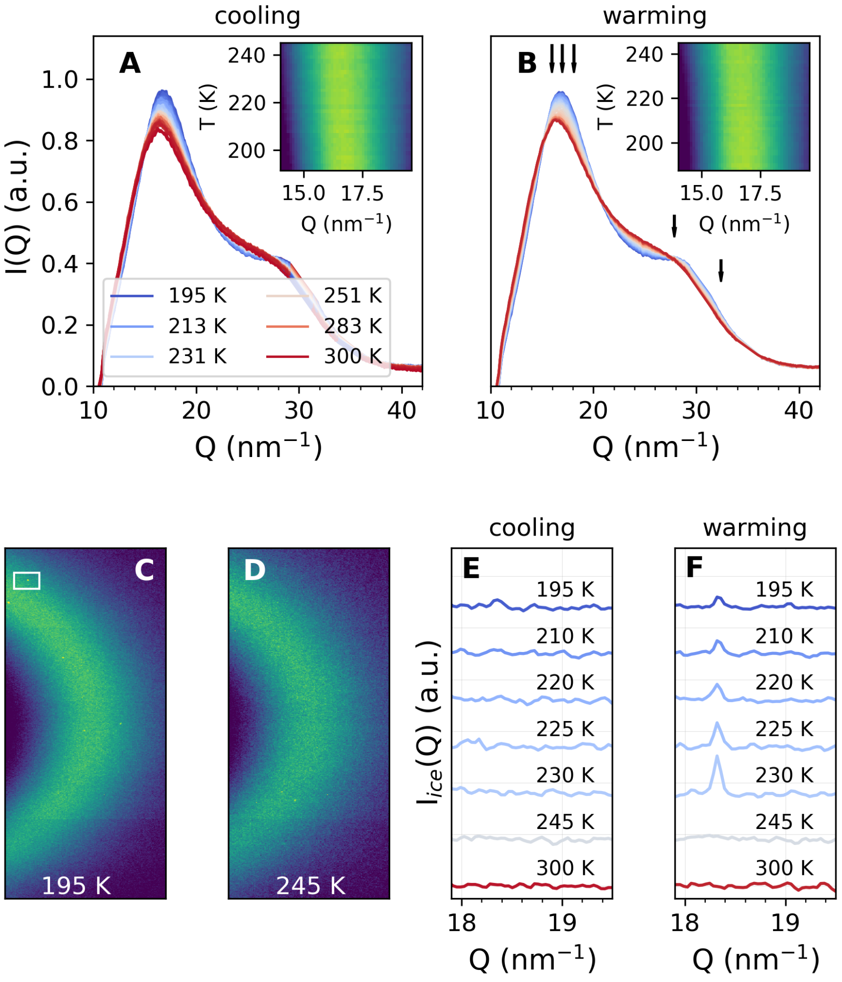

Figure S3: WAXS intensity measured for 10 mg/ml lysozyme in 23 mol% glycerol-water solution as a function of temperature while cooling down from $T = 300$ K to $T = 195$ K (panel A) and warming back up to room temperature (panel B). The insets in both panels represent the data in contour plots to emphasize that the ice peaks are absent in the cool down and manifest in the warm up. The black arrows in panel B indicate the peaks matching some of the Bragg peaks of hexagonal ice. (C, D) representative 2D scattering patterns measured upon heating at $T = 195$ K and $T = 245$ K, where ice peaks appear in the former case. The white rectangle highlights the ice peak which profile along the radial direction is plotted in panels E and F upon cooling and warming, respectively. In panels E and F, an offset has been added to facilitate the comparison between temperatures.

in 23 mol% glycerol-water solution. The similar behavior independent of the concentration of proteins in the system suggests that it mainly originates from the interplay of water and glycerol in the solvent itself.

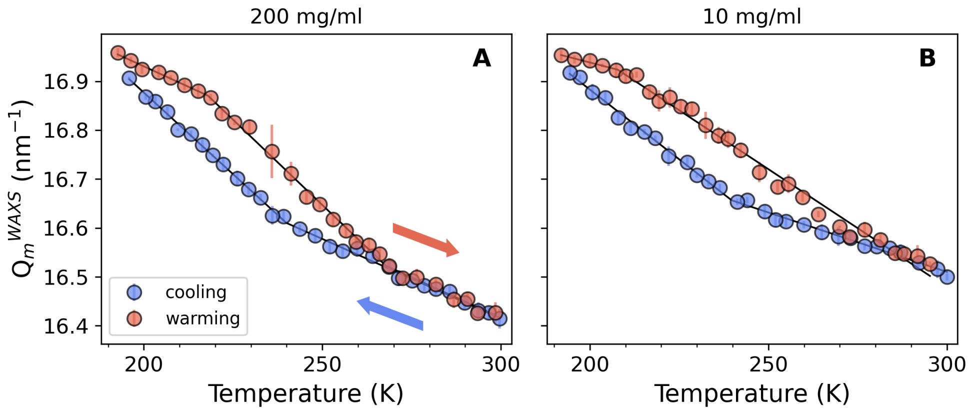

Figure S4: Temperature dependence of the Q-value of WAXS $I(Q)$ peak position during a temperature cycle for (A) 200 mg/ml, and (B) 10 mg/ml lysozyme in 23 mol% glycerol-water solution. The colors indicate measurements performed upon cooling (blue) or warming (red) as shown by the arrows.

Nanocrystallites in pure glycerol-water solution

As mentioned in the main text, the low temperature behavior in glycerol-water mixtures with concentrations of 15-28 mol% of glycerol likely stems from the formation of ice nanocrystallites occurring due to demixing of the solution components.[1–4] In Fig. S5, we present the measurement on a pure 23 mol% glycerol-water solution without proteins, where the formation of ice nanocrystallites can be expected. Indeed, as seen in panel A, upon reheating after cooling down to $T = 195$ K several small ice Bragg peaks are developed and can be resolved in the $I(Q)$ (Fig. S5 D).

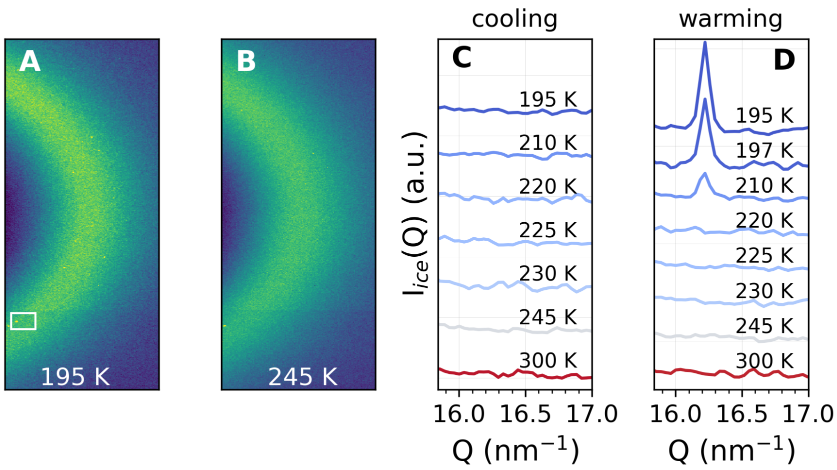

Figure S5: (A, B) representative 2D scattering patterns in the WAXS range measured on 23 mol% glycerol-water solution upon heating at $T = 195$ K and $T = 245$ K, where ice peaks appear in the former case. The white rectangle highlights the ice peak whose profile along the radial direction is plotted in panels C and D upon cooling and warming, respectively. In panels C and D, an offset has been added to facilitate the comparison between temperatures.

References

(1) Bachler, J.; Fuentes-Landete, V.; Jahn, D. A.; Wong, J.; Giovambattista, N.; Loerting, T. Glass polymorphism in glycerol–water mixtures: II. Experimental studies. *Phys. Chem. Chem. Phys.* **2016**, *18*, 11058–11068.

(2) Bachler, J.; Handle, P. H.; Giovambattista, N.; Loerting, T. Glass polymorphism and liquid–liquid phase transition in aqueous solutions: experiments and computer simulations. *Phys. Chem. Chem. Phys.* **2019**, *21*, 23238–23268.

(3) Hayashi, Y.; Puzenko, A.; Balin, I.; Ryabov, Y. E.; Feldman, Y. Relaxation dynamics in glycerol-water mixtures. 2. Mesoscopic feature in water rich mixtures. *J. Phys. Chem. B* **2005**, *109*, 9174–9177.

(4) Hayashi, Y.; Puzenko, A.; Feldman, Y. Ice nanocrystals in glycerol-water mixtures. *J. Phys. Chem. B* **2005**, *109*, 16979–16981.
